# Calcium imaging of CPG-evoked activity in efferent neurons of the stick insect

**DOI:** 10.1371/journal.pone.0202822

**Published:** 2018-08-24

**Authors:** Jens Goldammer, Charalampos Mantziaris, Ansgar Büschges, Joachim Schmidt

**Affiliations:** Animal Physiology, Institute of Zoology, Biocenter Cologne, University of Cologne, Cologne, Germany; Georgia State University, UNITED STATES

## Abstract

The stick insect is a well-established experimental animal to study the neural basis of walking. Here, we introduce a preparation that allows combining calcium imaging in efferent neurons with electrophysiological recordings of motor neuron activity in the stick insect thoracic nerve cord. The intracellular free calcium concentration in middle leg retractor coxae motor neurons and modulatory octopaminergic DUM neurons was monitored after backfilling lateral nerve nl5 that contains the axons of these neurons with the calcium indicator Oregon Green BAPTA-1. Rhythmic spike activity in retractor and protractor motor neurons was evoked by pharmacological activation of central pattern generating neuronal networks and recorded extracellularly from lateral nerves. A primary goal of this study was to investigate whether changes in the intracellular free calcium concentration observed in motor neurons during oscillatory activity depend on action potentials. We show that rhythmic spike activity in leg motor neurons induced either pharmacologically or by tactile stimulation of the animal is accompanied by a synchronous modulation in the intracellular free calcium concentration. Calcium oscillations in motor neurons do not appear to depend on calcium influx through voltage-sensitive calcium channels that are gated by action potentials because Calcium oscillations persist after pharmacologically blocking action potentials in the motor neurons. Calcium oscillations were also apparent in the modulatory DUM neurons innervating the same leg muscle. However, the timing of calcium oscillations varied not only between DUM neurons and motor neurons, but also among different DUM neurons. Therefore, we conclude that the motor neurons and the different DUM neurons receive independent central drive.

## Introduction

Two approaches allow recording of voltage changes across neuronal membranes: electrode-based techniques and optical measurements. An advantage of optical measurements over electrode-based techniques is that it is much easier to simultaneously measure activity in several neurons or at different locations of the same neuron. Changes in the concentration of intracellular free calcium ([Ca^2+^]_i_) in neurons that are detected by fluorescent calcium indicators are widely used as a proxy for neuronal activity [1–6]. For *in vivo* or *in situ* imaging of neuronal activity in locomotor systems genetically encoded Ca^2+^ indicators have been employed in a variety of animals like worms, flies zebrafish and mice [7–17]. However, this method is hardly applicable in animals with long reproduction cycles, e.g. in the large orthopteran insects like locusts and stick insects. Imaging of [Ca^2+^]_i_ in motor neurons in the ventral nerve cord has been performed in only a few studies on large insects (*Manduca sexta* [18]; Cricket [19]), in which intracellular electrodes were used for the application of a Ca^2+^ indicator. Here we sought to retrogradely load neurons with a Ca^2+^ indicator, similar to established procedures in vertebrates [20, 21]. Retrograde loading of stick insect efferent neurons with fluorescent dyes is well established [22]. Retrograde Ca^2+^ indicator fillings should allow simultaneous measurements of the electrical activity and [Ca^2+^]_i_ in efferent neurons in the stick insect locomotor system using optical and extracellular electrical recording techniques in a semi-intact preparation. Stick insects are successfully used for analyzing the mechanisms of the neural control of walking [23–26].

The functioning of locomotor systems in vertebrates and invertebrates depends on the coordinating output of central pattern generators (CPGs) located in the spinal cord or the ventral nerve cord, respectively. Generally, the outputs of CPGs allow for the proper sequential activation of motor neurons. Specifically, CPGs control the alternating activity in antagonistic motor neurons (for reviews see [27–30]). Sensory feedback from leg sense organs acts on CPGs, and thereby controls the relative phase and the magnitude of rhythmic locomotor neuron activity [31]. CPGs drive motor neurons that innervate leg muscles. In insects, each leg muscle can be innervated by 2 to about 25 excitatory motor neurons [22], and 1 to 2 inhibitory motor neurons [32, 33]. Insect leg muscles are also innervated by neuromodulatory octopaminergic DUM (dorsal unpaired median) neurons ([34, 35], reviews in [36, 37]). These efferent neurons have been shown to affect contraction properties of leg muscles, e.g. DUM cell activity increases and speeds up muscle contractions [38, 39]. In insects, however, the structures and functional properties of CPGs that drive leg motor neurons are not well known [24, 40].

In insects, CPGs driving leg motor neurons can be activated in the deafferented ventral nerve cord by application of muscarinic acetylcholine receptor agonists, such as pilocarpine. Pilocarpine application evokes alternating rhythmic activity in antagonistic leg motor neuron pools [41–44]. Pharmacological activation of cholinergic receptors by pilocarpine leads to a tonic membrane depolarization that is sculpted by recurrent inhibitory input into a rhythmic spike-burst pattern [45]. In leg motor neurons of the cricket, pilocarpine-evoked spike bursts were found to be correlated with [Ca^2+^]_i_ transients that were visualized by loading single motor neurons with the Ca^2+^ indicator Oregon Green BAPTA-1 via the intracellular electrode [19]. Data from locust and cockroach suggest that the fast tetrodotoxin-sensitive motor neurons spikes in insects themselves are Na^+^-dependent. [46–49]. In semi-intact stick insects, the amplitude of a tonic depolarization in mesothoracic motor neurons apparent during stepping of a single front leg on a tread mill was found to be Ca^2+^-dependent [26]. However, it is still unclear whether the increase in motor neuronal [Ca^2+^]_i_ depends only on spike activity or whether it is also related to subthreshold changes of the membrane potential.

Here, we sought to investigate, whether pilocarpine-evoked Ca^2+^ oscillations in motor neurons depend on action potential generation. For this investigation, we retrogradely filled the axons running through the lateral nerve 5 (nl5) with Oregon Green BAPTA-1. Nerve nl5 contains the axons of 22–24 retractor coxae motor neurons, the axon of the inhibitory neuron CI1 and the axons of 5–6 DUM neurons [22]. The neurites of these cells branch rather dorsally in the ganglion [22], allowing for optical monitoring [Ca^2+^]_i_, as a proxy for electrical activity in a semi-intact preparation.

## Material and methods

### Animals

Experiments were performed on adult female stick insects, *C*. *morosus* [50], from a colony maintained at the University of Cologne. Only female *C*. *morosus* were used because these stick insects are parthenogenetic animals that rarely produce males under good breeding conditions.

### Calcium imaging

#### Dissection and backfilling neurons for imaging experiments

Stick insects were fixed dorsal side up on a foam platform with dental cement (Protemp II, ESPE, Seefeld, Germany). All legs except the left front legs were amputated at their coxa–trochanteral joints. A preparation was established that allows backfilling axons in the left mesothoracic nerve 5 (nl5) with a Ca^2+^- indicator and subsequently optical recordings of the free intracellular Ca^2+^ concentration ([Ca^2+^]_i_) in the backfilled neurons of the mesothoracic ganglion. A lateral incision along the right body wall was made from the anterior mesothorax to the mid metathorax level. Two short transversal cuts at both ends allowed folding the dorsal cuticle aside and fixing it with minutien pins. To expose the mesothoracic ganglion the intact gut was moved to the right side. The body cavity was filled with saline (in mmol: NaCl 180; KCl 18; CaCl_2_ 8; MgCl_2_ 25; HEPES buffer 10; pH 7.2, after Weidler and Diecke [51]). The construction of the foam platform was such that the gut could be placed in a pool of saline to prevent it from drying out. Fat, connective tissue, and small tracheae that run into the coxa were carefully removed. The large longitudinal tracheae were left intact to allow respiration. During the preparations the body cavity was perfused with saline.

To gain access to nerve nl5 the tergo-sternal and the pleuro-sternal muscles were cut ventrally at their insertion. The tendons of the three large tergal retractor coxae muscles were also cut. The nl5 was cut before the branching point of the p branch, which innervates the pleural and tergal retractor muscles [52]. The cut end of nl5 was surrounded by a well of petroleum jelly (Weißes Vaselin, Medical Pharma, Bremerhaven, Germany). The well was then filled with distilled water and after five minutes replaced by a 5 mM solution of the high affinity Ca^2+^ indicator Oregon Green 488 BAPTA-1 dextran (OGB-1, 10.000 MW, Invitrogen, Eugene, OR) dissolved in distilled water. The well was covered with petroleum jelly and the animal was kept at room temperature for three to four hours in the dark to allow OGB-1 uptake. The stick insect saline was replaced several times during the diffusion of OGB-1. After dye diffusion, the Vaseline well and connective tissue dorsal to the ganglion were removed. The large longitudinal tracheae were cut anteriorly and were placed outside of the posterior end of the thoracic cavity to expose the ganglion. All nerves of the mesothoracic ganglion except for those used for electrophysiological recordings were crushed close to the ganglion with a pair of forceps to prevent feedback from sensory organs.

**Fig 1:**
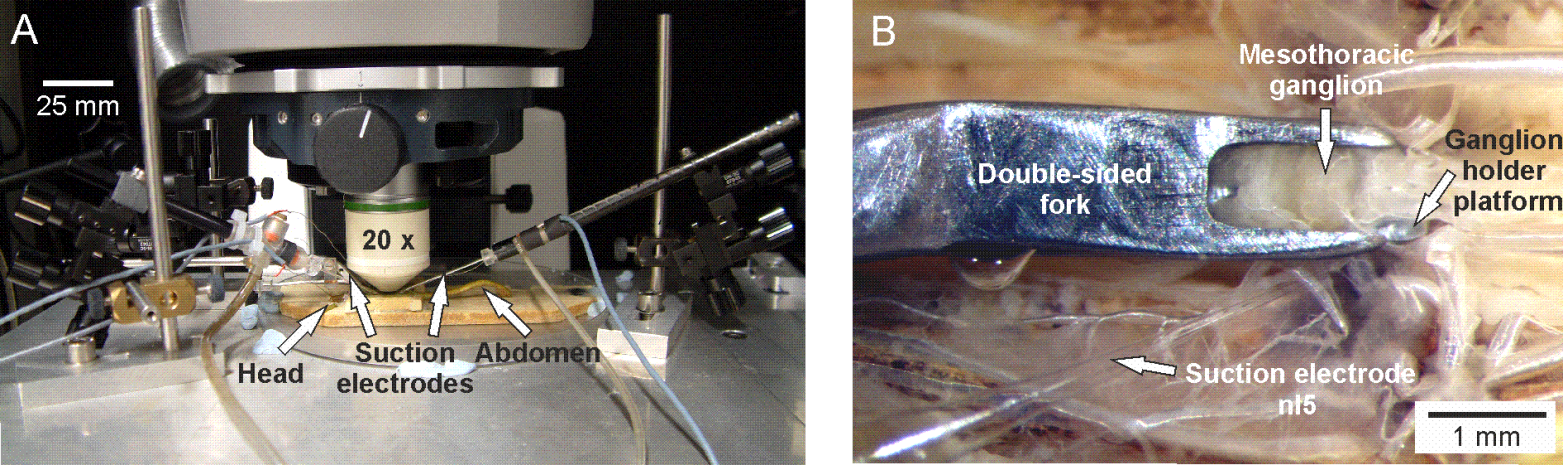
Experimental setup for imaging neuronal activity with a backfilled calcium indicator in combination with extracellular nerve recordings. (A) Overview of the experimental setup. Stick insect is mounted below the 20x imaging objective on an aluminum platform with two suction electrodes. (B) Dorsal view of the mesothoracic ganglion *in situ*. The mesothoracic ganglion lies ventral side down on a flat support surface and stabilized against movement by a double-sided fork placed dorsally on the lateral edges of the ganglion. A suction electrode records extracellularly electrical activity from the OGB-1-backfilled nl5 nerve stump that carries the axons of the retractor coxae innervating neurons.

To combine [Ca^2+^]_i_ imaging with extracellular nerve recordings, the foam platform with the animal was mounted on an aluminum platform (Fig 1A). The mesothoracic ganglion was stabilized in a steel screw clamp whose bottom portion was a flat support surface and whose upper portion consisted of a “U”-shaped fork that could be lowered so that the ganglion rested ventral side down on the flat support surface and was held in place from above by the fork (custom-built device, Electronics workshop, Zoological Institute, University of Cologne, Cologne, Germany). The side nerves and anterior connectives ran between the support surface and the fork (Fig 1B). This device allows imaging neurons through the dorsal surface of the ganglion. The steel screw clamp was attached to a micromanipulator (UN-3C, Narashige, Japan) that was mounted to the aluminum platform.

#### Pharmacological stimulation and backfilling of QX 314

Rhythmic motor activity in motor neurons and dorsal unpaired median (DUM) neurons was evoked by adding dropwise a 3 mM solution of pilocarpine (P6503, Sigma-Aldrich). To block intracellularly sodium channels we backfilled 5 mM OGB-1 mixed with different concentrations of the lidocaine derivate QX 314 (BIOTREND Chemicals AG, Wangen, Switzerland). The following concentrations of QX 314 were tested: 5 mM; 7 mM; 10 mM; 20 mM; 25 mM; 30 mM; 40 mM; and 100 mM. Effects on spike activity of the backfilled retractor coxae motor neurons (RetCx MNs) were first observed at 20 mM QX 314 and spike activity was completely abolished at QX 314 concentrations higher than 30 mM.

#### Electrophysiological recordings during Ca^2+^-imaging experiments

Custom-built suction electrodes with glass tips were used to record electrical activity in the backfilled nerve nl5 that contains the axons of retractor coxae motor neurons (RetCx MNs) and nerve nl2 that contains the axons of the antagonistic protractor coxae motor neurons (ProCx MNs). Glass tips of suction electrodes were manufactured from micropipettes (GB150 T-8P, Science Products, Hofheim, Germany) pulled on a vertical pipette puller Model 700C (David Kopf Instruments, Tujunga, California). Suction electrodes were attached to micromanipulators (UN-3C, Narashige, Japan) that were mounted on the aluminum platform.

Signals from suction electrodes were amplified (custom-built amplifier, model 102, Electronics workshop, Zoological Institute, University of Cologne, Cologne, Germany) 1000 times and filtered (lowcut 250 Hz, highcut 5 kHz). The data were digitized with a rate of 12.5 kHz (Micro 1401k II, Cambridge Electronic Design, Cambridge, UK) and stored on a personal computer using Spike 2 software (v7.01, Cambridge Electronic Design, Cambridge, UK).

#### Optical measurements

Monochromatic light at 496 nm (Polychrome V, Till Photonics, Gräfeling, Germany) was applied through the epifluorescence port of a Zeiss Axio Examiner D1 microscope (Carl Zeiss, Jena, Germany). Changes in fluorescence emission of OGB-1 were detected at 524 nm by a cooled charged-coupled device camera (AxioCam MRm, Carl Zeiss, Jena, Germany). A 20x W-Plan Apochromat water immersion objective (1.0 DIC, working distance: 1.8 mm, Carl Zeiss, Jena, Germany) was used for imaging regions of interest (ROI). Depending on the staining quality, the pixel resolution was set at 344 x 260 (binning factor 4 x 4) or 460 x 344 (binning factor 3 x 3) and the sensitivity of the AxioCam was frequently digitally enhanced. Video data acquisition was sampled at frequencies between 16 Hz and 35 Hz using AxioVision software (V4.8.2, Carl Zeiss, Jena, Germany). Optical recordings were pre-processed by using AxioVision software. Ca^2+^- imaging data were background corrected by subtracting values from a ROI placed on a part of the ganglion where no indicator staining was visible. For noise reduction, ratio-images were calculated by use of the confidence-mapping function [53] in the AxioVision software. All values given are changes in fluorescence divided by background intensity (ΔF/F0). Digitized imaging data were further processed with Excel (Microsoft).

#### Data analysis

For analyzing and displaying changes in [Ca^2+^]_i_, imaging data were either transferred from Excel into Spike 2 software to be combined with the digitized extracellular recordings or calcium data were displayed using IgorPro 6 software (Wavemetrics Inc., Lake Oswego, USA). Spike 2-software was also used to pre-process electrophysiological data for further analysis in MATLAB (vR2011b; The MathWorks, Inc., Natick, USA). To correlate neuronal activity with changes in [Ca^2+^]_i_, the gross neuronal activity was estimated by rectifying and smoothing (first order low pass filter, time constant 50 ms) the waveforms of extracellular recordings. Custom written SPIKE2 scripts were used to mark bursts of action potentials, according to the following burst criteria: maximum initial interval signifying burst onset of 0.08 s; inter-spike interval of 0.2 to 0.4 s; minimum number of four spikes in a burst; minimum burst duration of 0.3 s; and minimum inter-burst interval of 0.4 s.

To compare extracellularly recorded firing of action potentials with corresponding [Ca^2+^]_i_ transients in selected regions of interest (ROIs) custom written scripts in MATLAB were used. Burst intensities were measured by determining the integrals of motor neuron activity transients in rectified and smoothed recording traces within the intervals between the burst onsets and offsets. These intervals were also used to calculate the integrals under the [Ca^2+^]_i_ elevations. To calculate the [Ca^2+^]_i_ integrals and to compensate for changes in fluorescence due to dye bleaching, the measured Ca^2+^- transients were median filtered with a long time constant (28 s to 64 s) and filtered values were subtracted from measured Ca^2+^ transients. Therefore, [Ca^2+^]_i_ transients are slightly smaller than in the original traces. The integrals of burst activities and of [Ca^2+^]_i_ transients were normalized by dividing the corresponding burst duration in order to calculate the mean integrals independent of burst duration. A regression analysis in MATLAB was used to analyze linear correlations between the integrals of mean burst activities and [Ca^2+^]_i_ integrals. Correlation coefficients were regarded as statistically significant when P<0.001.

For testing differences in periods of rhythmic burst activity in motor neurons and DUM neurons a t-test assuming different variances was performed in EXCEL (Microsoft). Final figures were prepared in Corel Draw (Version 13, Corel, Ottawa, CA).

### Intracellular recordings

#### Preparation and pharmacological stimulation

Stick insects were glued to a foam platform and all legs were amputated as described above. The thorax was opened to allow access to the mesothoracic ganglion and nerves nl5 and nl2. The gut was moved aside, and connective tissue was carefully removed to expose the mesothoracic ganglion. The connectives between the prothoracic and the mesothoracic ganglion and between the mesothoracic and the metathoracic ganglion were cut along with all side nerves of the mesothoracic ganglion, except for nl2 and nl5 on the right side.

#### Intracellular recordings and injection of QX 314

To perform intracellular recordings of motor neuron activity, the mesothoracic ganglion was placed on a wax-covered steel platform and pinned down with cactus spines (*Nopalea dejecta*) according to established procedures [54]. To facilitate electrode penetration, crystals of a proteolytic enzyme (Pronase E, Merck, Germany) were placed directly on the ganglionic sheath before filling the body cavity with saline. To stop the effect of the enzyme the ganglion was rinsed with saline after 40 seconds. Finally, the body cavity was filled with saline Sharp microelectrodes of 20–30 MΩ resistance were made of borosilicate glass (GB150 TF-8P, Science Products, Hofheim, Germany) using a micropipette puller (P-1000, Sutter Instruments). Electrodes were filled with 3 M KAc/0.1 M KCl electrolyte solution.

For intracellular injection of QX 314 (BIOTREND Chemicals AG, Wangen, Switzerland) into RetCx MNs, QX 314 was added to the el ectrolyte solution up to a final concentration of 0.1 M. In one of seven experiments depolarizing pulses (1–2 nA, 300 ms duration, 1 Hz frequency) were used to inject QX 314. Spikes were blocked after 18 minutes. In all other experiments constant currents (1 – 2nA) were used to inject QX 314. During current injection, depolarizing pulses with varying amplitudes and durations (300 ms—1 s) were applied about every 10 s to test whether spike generation was blocked. (see also the Results section).

RetCx MN activity was recorded from neuropilar processes. RetCx MNs were identified by a one-to-one relationship between spikes in the intracellular recording and spikes in the extracellular nl5 recording. Signals were recorded in bridge mode (intracellular amplifier SEC-10L, npi electronics, Tamm, Germany). The data were digitized at a rate of 6.5 kHz (Micro 1401k II, Cambridge Electronic Design, Cambridge, UK) and stored on a personal computer using Spike 2 software.

### Extracellular recordings

Activity in RetCx MNs and ProCx MNs was recorded extracellularly from nerves nl5 and nl2 respectively, using hook electrodes [55]. Signals were amplified 1000 times, filtered (lowcut 200 Hz, highcut 3 kHz), and digitized using the same equipment as described for suction electrode recordings (see above).

#### Data analysis and display

Spike 2-software was used to display recorded data. Final figures were prepared in Corel Draw (for details see above).

## Results

In the stick insect, mesothoracic leg nerves nl5 were backfilled with the Ca^2+^ indicator OGB-1 to study *in situ* the free intracellular Ca^2+^ concentration ([Ca^2+^]_i_) in retractor coxae motor neurons and neuromodulatory dorsal unpaired median (DUM) neurons that were stimulated pharmacologically [41] or by stimulation of mechanosensors on the animals body [56]. After backfilling nerve nl5 the animal was placed under the microscope and the filled nerve was sucked into a suction electrode.

### Ca^2+^-transients in leg motor neurons during pharmacologically-evoked rhythmic activity

**Fig 2:**
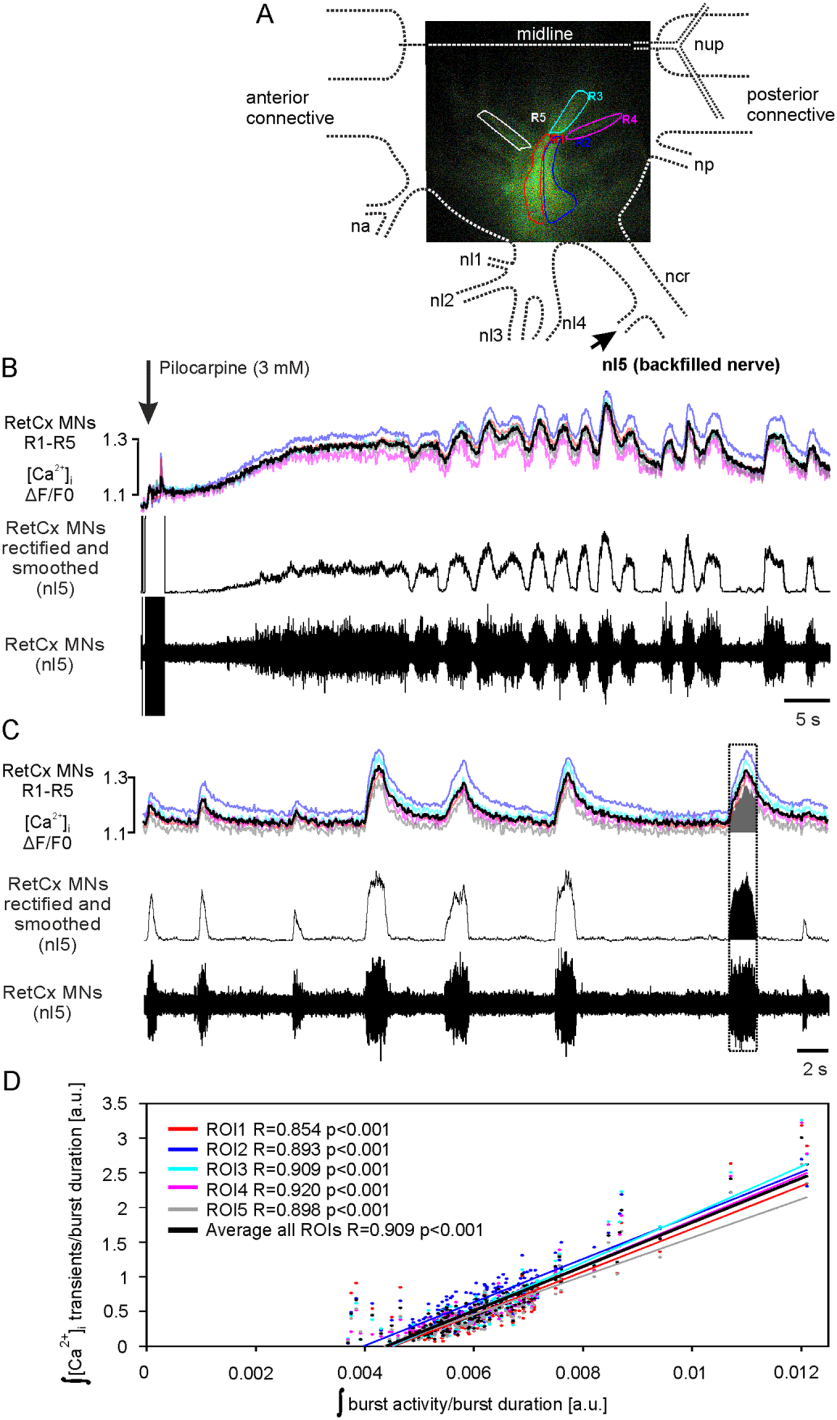
Pilocarpine-induced [Ca^2+^]_i_ elevations in RetCx MN neurites and extracellular recorded spike activity of RetCx MNs. (A) [Ca^2+^]_i_ was measured in five regions of interest. Dashed lines indicate the outline of the respective hemiganglion and nerve roots. na nervus anterior, nl1-nl5 nervi laterali 1 to 5, ncr nervus cruris, np nervus posterior, nup nervus unparis (see [22] for details). The arrow indicates the backfilled nerve nl5 that contains the axons of retractor motor neurons (RetCx MNs). (B) Application of pilocarpine (arrow) evokes tonic firing of retractor coxae MN (RetCx) units followed by rhythmic bursting (bottom trace). The middle trace shows the rectified and smoothed RetCx spike activity, which is accompanied by an increase in [Ca^2+^]_i_ across five measured ROIs (upper trace; colors match ROIs R1-R5 as shown in upper right inset displaying ROIs placed over stained MN structures; black trace shows average of colored traces). (C) Time course after established pilocarpine-induced rhythmicity. During bursts of spikes (bottom trace), the [Ca^2+^]_i_ in RetCx MN neurites increases (upper trace, ROI colors as in (A). Note that [Ca^2+^]_i_ transients across all ROIs are similar in shape compared to rectified and smoothed extracellular RetCx MN activity (middle trace). Black trace shows average of colored traces. Black and gray shaded areas in boxed area illustrate measurements of integrated activity used for (D). (D) Integrated [Ca^2+^]_i_ divided by burst duration over integrated burst activity divided by burst duration shows linear relationships for all ROIs. Black line shows average of colored lines. Normalized data are shown as arbitrary units (a.u.).

The muscarinic ACh receptor agonist pilocarpine activates CPGs in thoracic ganglia of insects that generate rhythmic alternating activity in antagonistic motor neurons [41]). Fig 2B and 2C are typical examples of five experiments showing the development in [Ca^2+^]_i_ and spike activity in RetCx MNs after application of pilocarpine (3 mM). Five regions of interest (ROIs R1-R5; see Fig 2A) were defined, located mainly over the primary neurites (ROIs R1 –R3) and bundles of secondary neurites branching off in three different regions of the motor neurons (ROIs R4 and R5). As all RetCx MNs have overlapping neurites with similar branching patterns [22], [Ca^2+^]_i_ measured in these regions is the integrated activity of many MNs. Around 10 seconds after application of pilocarpine changes in relative fluorescence (ΔF/F0) in all five ROIs became apparent (see colored traces in Fig 2B, colors correspond to respectively colored borders of ROIs). After about 30 s the increased [Ca^2+^]_i_ started oscillating. Changes in [Ca^2+^]_i_ were temporally uniform across all ROIs (see also at an expanded time scale in Fig 2C).

The increase in [Ca^2+^]_i_ and the oscillations corresponded to the spike activity in RetCx MNs as seen in the middle traces in Fig 2B and 2C. The lower trace in (B) shows the initial firing of small amplitude spikes and the successive recruitment of larger amplitude spikes. The tonic firing developed into a bursting pattern that corresponds to the Ca^2+^ oscillations, which is clearly visible in Fig 2C at an extended time scale. The middle trace shows an integral of the MN activity that was rectified and smoothed. In all five ROIs the integrals of the Ca^2+^_i_ transients (measure from start to end of a burst) show a linear correlation with the corresponding burst activity (R = 0.854 to 0.920, p<0.001; Fig 2D; S1 Data). Linear correlation of [Ca^2+^]_i_ transients and motor neuron activity was observed in all experiments that were evaluated (N = 6), with the exception of very small [Ca^2+^]_i_ transients close to the noise level. Thus, the changes in [Ca^2+^]_i_ mirror the overall spike activity in the respective bursts, suggesting that _i_ Ca^2+^ influx into motor neurons depends on spike activity.

### Intracellular Ca^2+^ activity in motor neurons evoked by tactile stimulation

In stick insects, tactile stimulation of the abdomen can evoke short bouts of alternating activity in antagonistic leg motor neurons of deafferented thoracic ganglia reflecting functions of the locomotor system [56, 57]. Evoked by pilocarpine or by tactile stimulation, spike bursting in a motor is based on the same two mechanisms, a tonic depolarization and phasic synaptic inhibition [45, 57].

**Fig 3:**
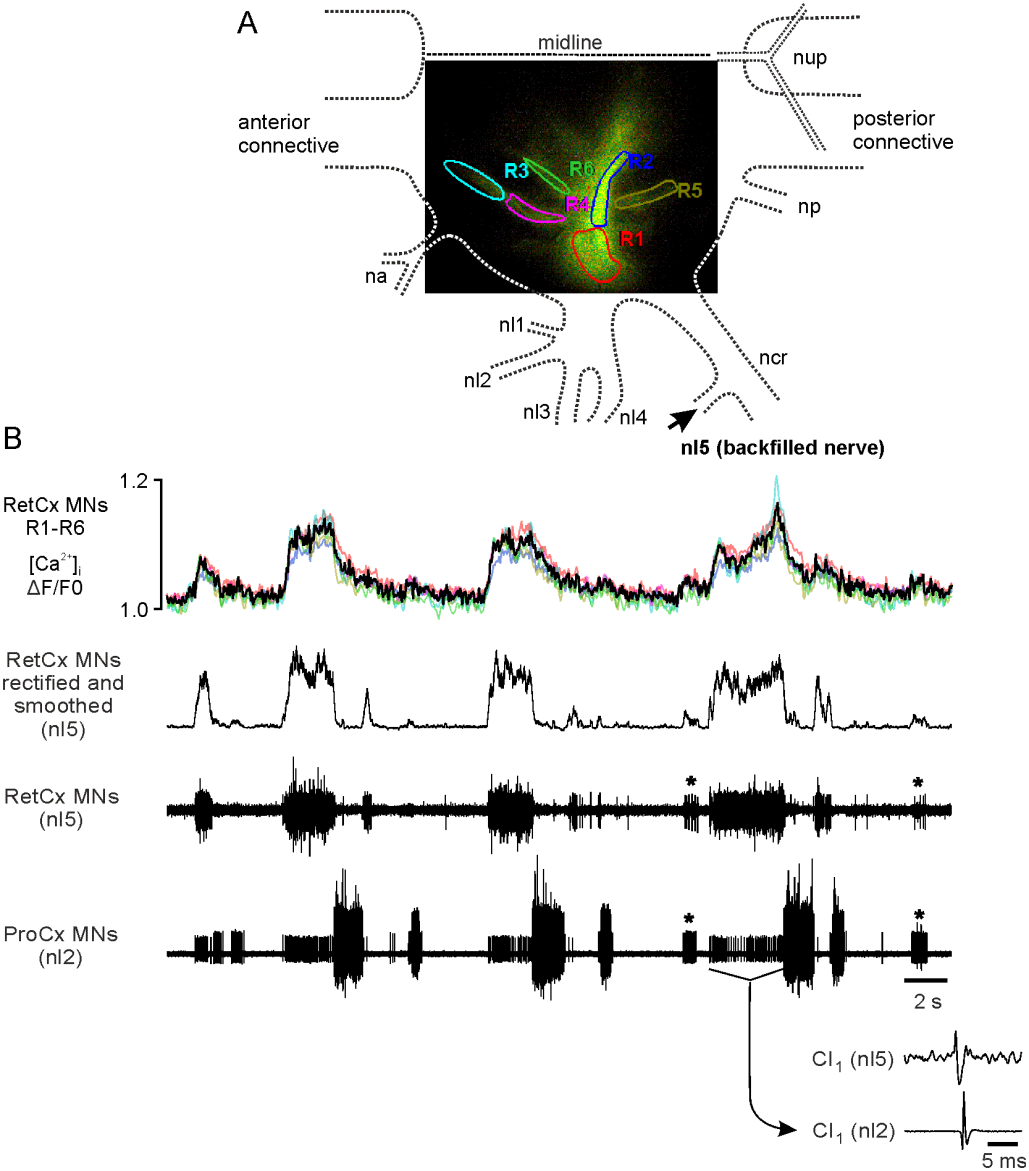
[Ca^2+^]_i_ elevations in RetCx MN neurites elicited by tactile stimulation of the animal’s abdomen. (A) [Ca^2+^]_i_ was measured in six regions of interest. Dashed lines indicate the outline of the respective hemiganglion and nerve roots (see Fig 2 for details). (B) Tactile stimulation elicited [Ca^2+^]_i_ transients in RetCx MN neurites (first trace, colors match ROIs R1-R6 as shown in (A); black trace shows average of colored traces) in-phase to bouts of RetCx bursts (third trace)). [Ca^2+^]_i_ transients across all ROIs are similar in shape compared to rectified and smoothed extracellular RetCx MN activity (second trace). Tactile stimulation evoked switching of activity from RetCx MN bursts to ProCx MN bursts (fourth trace). Asterisks mark co-activated small MN units apparent in both recordings. The smallest units in the nl2 recording are spikes generated by the common inhibitor neuron CI1 because these spikes can be also detected in the nl5 recording as shown in an average of 94 sweeps triggered by the spikes in nl2 (inset below).

To elucidate whether bursting activity in RetCx MNs that is evoked by tactile sensory stimulation is also accompanied by an increase in [Ca^2+^]_i_ we stimulated the abdomen of stick insects (N = 12) with a paint brush. Switching of activity between the antagonistic RetCx and ProCx MNs upon tactile stimulation of the abdomen of the animal is shown in Fig 3. The experiment shows that Ca^2+^ transients in RetCx MNs reflect RetCx spike activity similar to experiments in which pilocarpine was used to elicit alternating activity in leg motor neurons. Thus, Ca^2+^ transients in motor neurons during switching are generated regardless of whether alternating activity is evoked pharmacologically or by tactile stimulation.

### Ca^2+^ oscillations and their dependence on spike activity

As noted above, Ca^2+^ transients reflect spike bursting of the respective RetCx MNs recorded from nerve nI5. It is common knowledge that action potentials open voltage-sensitive Ca^2+^ channels in neurons. In insect motor neurons, however, subthreshold depolarizing current injections [19] and stimulation of nicotinic ACh receptors [58] may also cause an increase in [Ca^2+^]_i_. Therefore, through blocking of spike generation in RetCx MNs, we tried to find evidence that Ca^2+^ enters the RetCx MNs through low-voltage-activated and/or ligand-gated Ca^2+^ channels. To this end, the lidocaine derivate QX 314, a blocker of voltage sensitive Na^+^-channels [59, 60], was injected intracellularly into RetCx MNs.

**Fig 4:**
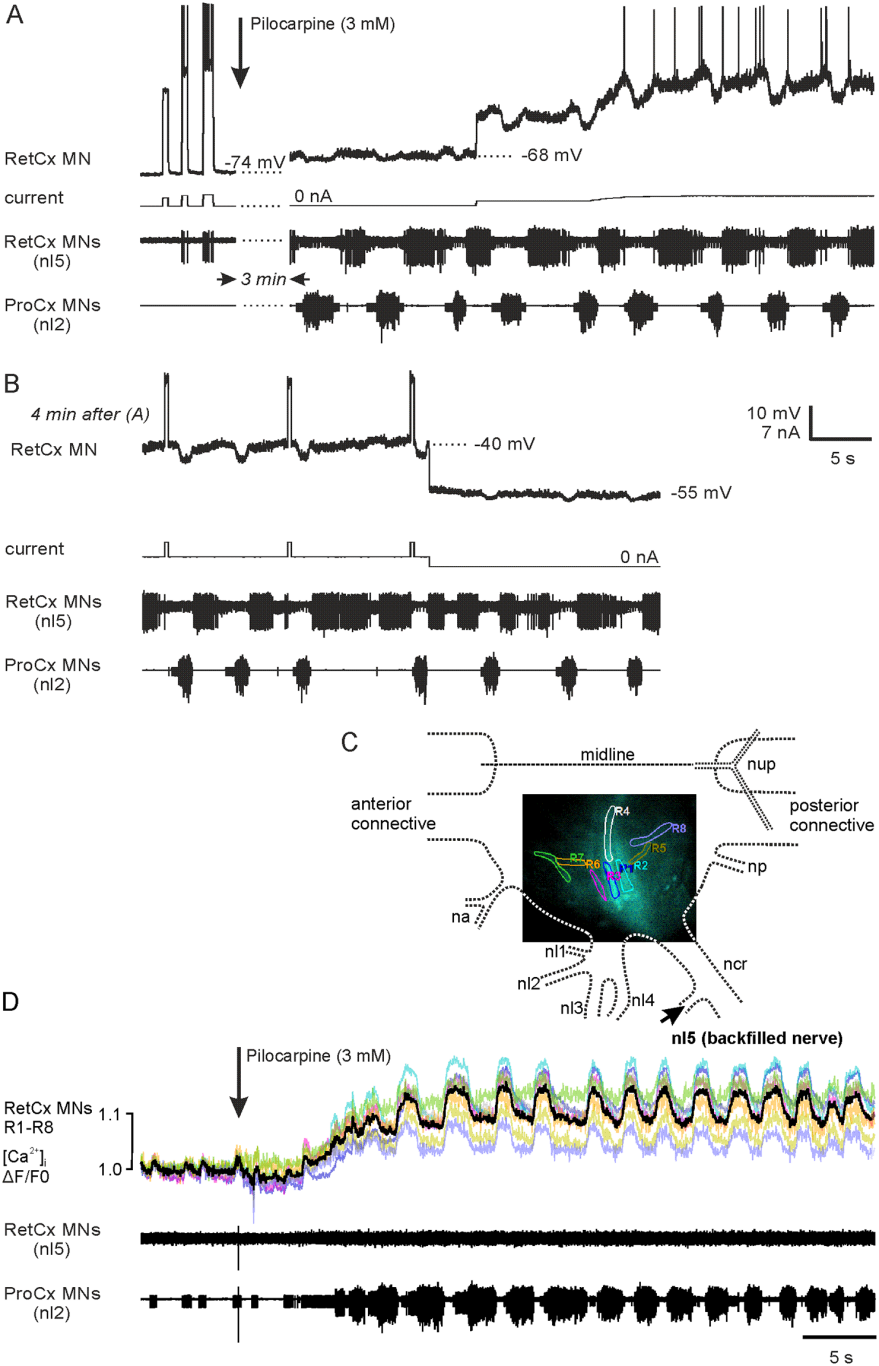
Pilocarpine-evoked membrane potential oscillations in RetCx MNs do not depend on voltage-gated sodium currents. (A) Simultaneous intracellular recording of RetCx MN membrane potential and extracellular spike activity of the ipsilateral antagonistic retractor and protractor MN pools (RetCx MNs (nl5) and ProCx MNs (nl2)). Recording electrodes were filled with the lidocaine derivative QX 314 and rhythmic activity was induced by application of pilocarpine. Spike activity in the RetCx MN is visible upon depolarization in the intracellular and extracellular recording traces before application of pilocarpine. Three minutes after pilocarpine application, the membrane potential of the RetCx MN is depolarized and oscillates, and antagonistic ProCx MNs are active in alternation (extracellular traces). QX 314 is injected into the neuron by application of positive current. (B) The same recording as in (A) after four minutes. Spikes cannot be generated by current injection anymore and thus have been blocked, whereas membrane potential oscillations persist. (C) [Ca^2+^]_i_ was measured in eight regions of interest. Dashed lines indicate the outline of the respective hemiganglion and nerve roots (see Fig 2 for details). (D) [Ca^2+^]_i_—oscillations in RetCx MN neurites persist when spike activity is blocked by QX 314. In this experiment 30 mM QX 314 was backfilled together with the calcium indicator OGB-1 into RetCx MN neurites. Application of pilocarpine (arrow) evokes ProCx nerve activity (bottom trace), and alternating [Ca^2+^]_i_ transients in RetCx MN neurites (upper trace, colors match ROIs 1–8 as shown in (C); black trace shows average of colored traces), while no spikes could be recorded from RetCx MN axons in nerve nl5 (middle trace).

We tested the effect of QX 314 on spike generation and membrane potential oscillations in RetCx MNs with intracellular recordings of the membrane potential. For drug application, electrodes were filled with standard 3 mM KAc/100 mM KCl solutions containing 100 mM QX 314. QX 314 was injected into RetCx MNs using constant depolarizing currents of 1–2 nA. In all seven preparations, injection of QX 314 caused a gradual decrease in spike amplitude after some minutes and blocked spike activity altogether on average after 10 minutes. A typical experiment is shown in Fig 4A and 4B. Injection of 2 nA depolarizing current pulses evoked spike activity in the RetCx MN, also visible in the extracellular recording trace (RetCx MNs (nl5)). Application of pilocarpine (0.1–0.2 mM) caused alternating activity in RetCx und ProCx MNs and membrane potential oscillations in the intracellularly recorded RetCx MNs. In four of seven experiments membrane potentials of RetCx MN could be compared before application of pilocarpine and in the presence of pilocarpine. (In the other three experiments RetCx MNs were impaled after application of pilocarine.) The membrane potential gradually depolarized from -72.6 ±4.1 before application of pilocarpine to -66.1 ±3.6 mV after alternating rhythmic activity in RetCx MNs and ProCx MNs was established. Based on the relatively negative resting membrane potential of -74 mV, we assume that the RetCx MN in Fig 4 was a fast motor neuron [61]. Spike activity in ProCx MNs was accompanied by membrane hyperpolarizations in the RetCx MN, most obvious in Fig 4A upon motor neuron depolarization by current injection. When starting constant current injection about five minutes after impalement the neuron generated spikes with a lower frequency than shortly after impalement (see response to current pulses at the start of the trace). This observation might indicate that QX 314 already leaked into the cell. Four minutes later (Fig 4B) spike generation by current injection was not possible any more. At this stage, pilocarpine-evoked oscillations had amplitudes in the range of 1.5 to 9 mV (5.1 ± 2.9 mV) in seven experiments (S2 Data).

Spike bursting in other RetCx MNs that were not impaled by the QX 314 electrode was retained. In three control experiments, RetCx MNs were recorded with electrodes containing 3 M KAc/100 mM KCl without QX 314. After injection of constant depolarizing currents between 2–4 nA for an average time of 12 min there was no sign of decrease in spike amplitude or even blocking of the generation of action potentials (S1 Fig). These experiments demonstrate the effectivity of QX 314 as an intracellular blocker of spike activity in RetCx MNs.

To investigate the role of action potential generation on [Ca^2+^]_i_ transients QX 314 was backfilled into RetCx MNs together with the Ca^2+^ indicator OGB-1 for three to four hours prior to the experiment in order to investigate whether Ca^2+^ oscillations may persist when spike activity is blocked in RetCx motor neurons. Fig 4D shows that application of 3 mM pilocarpine evoked Ca^2+^ oscillations in RetCx MNs, whereas no spikes could be recorded from nerve nl5. Again, Ca^2+^ oscillations were temporally uniform across all ROIs. In contrast to RetCx MNs, ProCx MNs generated spike bursts appearing in alternation with the periodic increases in RetCx MNs [Ca^2+^]_i_. These results were obtained in a total of nine experiments indicating that Ca^2+^-oscillations in RetCx MNs are independent of recurrent spike bursts in these neurons. Thus, Ca^2+^ probably also entered the motor neurons under investigation though low-voltage-activated and ligand-gated Ca^2+^ channels (see Discussion).

### Intracellular Ca^2+^ activity in dorsal unpaired median neurons and motor neurons

**Fig 5:**
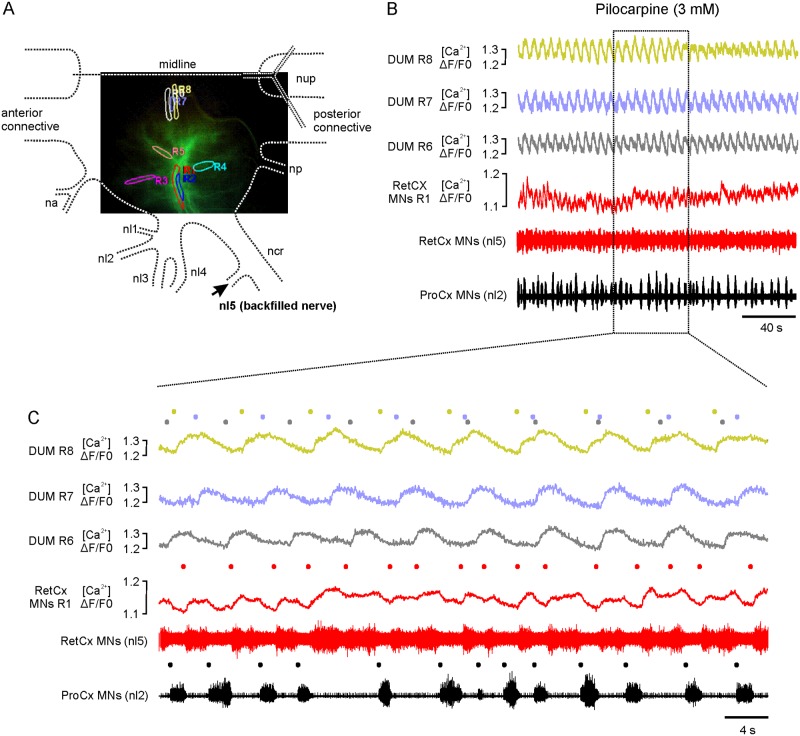
Pilocarpine-induced [Ca^2+^]_i_ transients in lateral DUM neurites. (A) [Ca^2+^]_i_ was measured in eight regions of interest (RetCx MNs ROIs R1-R5, lateral DUM neurites ROIs R6-R8). Dashed lines indicate the outline of the respective hemiganglion and nerve roots (see Fig 2 for details). (B) Traces 1–3: [Ca^2+^]_i_ transients in DUM ROIs 6–8; trace 4: [Ca^2+^]_i_ transients of MN ROI 1; trace 5: extracellularly recorded RetCx nerve activity, and trace 6: extracellular recorded ProCx nerve activity. (C) Magnification of boxed area in (B). Gray, yellow, and blue dots indicate start points of respective colored [Ca^2+^]_i_ transients in DUM neurites. Please note changes in relative position indicating shifting phases. Red dots indicate start points of respective colored [Ca^2+^]_i_ transients in RetCx MNs. Black dots indicate start points of bursts in RetCx MNs.

As described above, all excitatory RetCx motor neurons showed oscillations in [Ca^2+^]_i_ along with their burst activity following either pharmacological or tactile activation of neurons in the locomotor system. As reported above, nerve nl5 also contains the axons of 5–6 dorsal DUM neurons [22]. Four of these are known to have axons in other ipsilateral leg nerves of the ganglion as well [62]. We were interested in whether DUM neurons show Ca^2+^ oscillations and whether changes in [Ca^2+^]_i_ in different DUM neurons differ. In five animals, [Ca^2+^]_i_ was measured in primary neurites of DUM neurons, as cell bodies were not sufficiently dye-filled to provide a satisfying signal-to-noise ratio. Because DUM cell bodies lie close to the midline of the ganglion, signals from primary neurites originating from the cell body can be measured in regions with rather sparse motor neuron branching, and therefore there is little risk of contamination by motor neuron fluorescence. The typical spacing of regions of interest can be seen in Fig 5A. RetCx MN [Ca^2+^]_i_ was measured in ROIs R1 to R5 and DUM [Ca^2+^]_i_ in regions R6 to R8.

**Fig 6:**
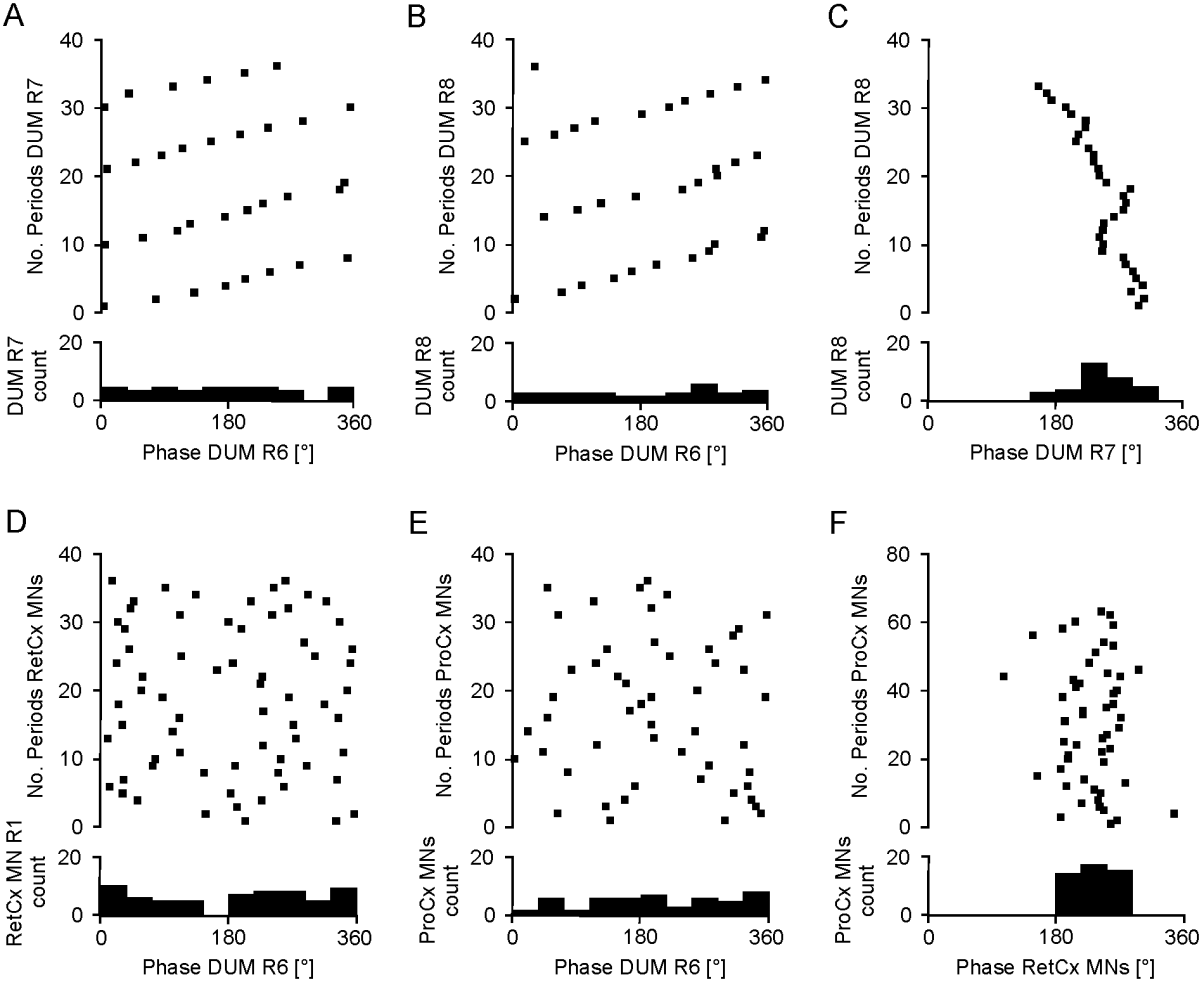
Pilocarpine-induced [Ca^2+^]_i_ transients in different DUM neurons are not phase-locked. (A) [Ca^2+^]_i_ transients in DUM ROI R7, as indicated by numbers of periods, start progressively later in the phases of DUM ROI R6. (B) [Ca^2+^]_i_ transients in DUM ROI R8 start progressively later in the phases of DUM ROI R6. (C) [Ca^2+^]_i_ transients in DUM ROI R8 start progressively earlier in the phases of DUM ROI R7. (D) [Ca^2+^]_i_ transients in RetCx MN ROI R1 do not show regular shifting in phases or phase locking in relation to DUM ROI R6 activity. (E) Spike bursts in ProCx MNs do not show regular shifting in phases or phase locking in relation to DUM ROI R6 activity. (F) Spike bursts in ProCx MNs are generally coupled to RetCx MN ROI R1 phases in the range between 180°–300° (S3 Data).

Fig 5B and 5C (three upper traces) show typical features of changes in DUM [Ca^2+^]_i_ after application of pilocarpine (3 mM). After application of pilocarpine, [Ca^2+^]_i_ in DUM neurons oscillated. Oscillation periods varied between about 4.9 s and 9.7 s between DUMs in four different animals (S4 Data). Oscillation periods also varied among different DUM neurons in the same animal, although, in a much smaller range. Periods in DUM R6 were 5.9 ±0.26 s (n = 35), in DUM R7 6.6 ±0.15 (n = 33), and 6.4 ±0.22 (n = 33) in DUM R8. All differences were highly significant (p≤0.001) or significant for DUM R7 vs. DUM R8 (p≤0.05) (S3 Data). Another feature seen in this recording is a shifting of periods between DUM R6 and R7 and DUM R6 and R8 (see dots indicating onset of Ca^2+^ transients in Figs 5C, 6A and 6B). Shifting between DUM R7 and DUM R8 was less pronounced (Fig 6C). The different mean periods and the phase differences between Ca^2+^ oscillations indicate that these signals actually correspond to recordings from three independent oscillating DUM neurons. Similar phase shifting was observed in a second recording. In three other experiments we either observed in phase oscillations in all ROIs (N = 2) or neither phase coupling nor shifting (N = 1).

Average oscillation periods in RetCx MNs ranged from 1.6 s to 4.8 s in different experiments and were about 39 to 68% shorter than the oscillation periods in DUM neurons in the same experiment (N = 4) (S4 Data). No phase coupling between Ca^2+^ oscillations in DUM neurons and RetCx MNs or ProCx MN oscillatory activity was found (N = 4), as shown for DUM R6 in Fig 6D and 6E. In contrast, onsets of spike bursts of ProCx MNs appeared mostly in a range of 180° to 290° in the RetCx MN activity cycles (Fig 6F).

## Discussion

In this study we have introduced a preparation that allows electrophysiological recordings of motor activity together with Ca^2+^ imaging in the stick insect locomotor system. Firstly, we show that rhythmic activity in leg motor neurons induced by pharmacological activation of CPGs or switching spike burst acitivity in antagonistic motor neurons evoked by tactile stimulation of the experimental animal is accompanied by a synchronous modulation in [Ca^2+^]_i_ leg motor neurons as monitored by Oregon Green BAPTA-1 that was retrogradely filled into the motor neurons. Interestingly, rhythmic modulation in [Ca^2+^]_i_ was also apparent in the modulatory DUM neurons innervating the leg muscles, however, with a different timing of oscillations. Secondly, rhythmic changes in [Ca^2+^]_i_ in motor neurons do not appear to depend only on Ca^2+^ influx through voltage sensitive Ca^2+^ channels that are gated by action potentials as revealed by pharmacologically blocking action potentials in the motor neurons.

### Ca^2+^ oscillations in leg motor neurons

The muscarinic acetylcholine receptor agonist pilocarpine is known to activate central pattern generating networks (CPGs) in locomotor systems in stick insects [41] and other arthropods [43, 44, 63]. In stick insect RetCx MNs, the application of pilocarpine led initially to tonic spike activity with an increasing recruitment of larger units. After a few seconds, the tonic activity developed into a pattern of spike bursts that occurred in anti-phase to the rhythmic activity of the antagonistic ProCx MNs. These changes fit the observation by Büschges [45], who recorded stick insect tibial motor neurons intracellularly and showed that application of pilocarpine on the ventral nerve cord leads to a membrane depolarization that eventually is sculpted into a rhythmic activity pattern by recurrent inhibitory input. The tonic activity increase and the bursting pattern of the RetCx MNs were reflected by an increase in [Ca^2+^]_i_ and subsequent Ca^2+^ oscillations in the motor neurons. Therefore, the oscillations in [Ca^2+^]_i_ in RetCx MNs are likely to derive from the membrane depolarization that develops into bursting activity by inhibitory CPG input and opens voltage-sensitive Ca^2+^ channels. Oscillations in [Ca^2+^]_i_, however, appear not to depend entirely on spike activity that opens high-voltage activated Ca^2+^ channels as blocking the generation of spikes with QX 314 did not block Ca^2+^ oscillations. These observations are consistent with findings on cricket motor neurons in which sub-threshold current injections cause increases in [Ca^2+^]_i_ activity [19]. These authors speculated that Ca^2+^ may enter the cell through low-threshold activated Ca^2+^ channels. This speculation is supported by our QX 314 experiments and by the finding that subthreshold Ca^2+^ potentials and low threshold Ca^2+^ currents have not only been observed in vertebrate motor neurons [64, 65] but also in insect motor neurons [66, 67].

It is possible that in addition to voltage-gated channels also ligand-gated channels allow Ca^2+^ entry into motor neurons because application of ACh onto stick insect motor neuron cell bodies was reported to evoke an inward current through nicotinic receptors mediated by about 18% by an influx of Ca^2+^ [58]. The authors also showed that in contrast to nicotinic agonists, application of pilocarpine onto motor neuron cell bodies did not affect the membrane potential. Therefore, it is unlikely that pilocarpine is a direct mediator of Ca^2+^ entry at the motor neuron membrane. Another possible source of Ca^2+^ entry into the cytosol is from internal stores [68]. In insects, Ca^2+^ induced Ca^2+^ release has been shown in photoreceptor cells [69, 70] and in efferent DUM neurons of cockroach [71]. In the locust, calcium release from intracellular stores via phospholipase C and inositol 1,4,5-trisphosphate receptor activation has been shown [72].

In our experiments, we did not detect any segments of RetCx motor neurons that did not exhibit oscillations in [Ca^2+^]_i_. In addition, the oscillations were temporally uniform across all ROIs. Even though the approach of measuring the integrated [Ca^2+^]_i_ in potentially 22–24 RetCx motor neurons [22] in a non-cleared ganglion and highest sampling frequencies of 35 Hz naturally limits the spatial and temporal resolution in our recordings, the observations indicate a rather wide distribution of low-voltage activated Ca^2+^ channels over the neurites of the motor neurons. Our results corroborate similar observations in single cricket motor neurons [19] and heart beat interneurons in the leech [73].

### Functional implications of Ca^2+^- entry into motor neurons

The functional relevance of Ca^2+^ oscillations in motor neurons during pilocarpine evoked CPG activity becomes apparent when considering that the level of [Ca^2+^]_i_ determines the level of depolarization in motor neurons during walking [26]. In a single leg preparation of the stick insect, treadmill stepping of a front leg evokes a tonic depolarization in ipsilateral middle leg motor neurons [74]. This depolarization decreases when the cell is recorded intracellularly with an electrode filled with the calcium chelator BAPTA [26]. It is quite conceivable to assume that the tonic membrane depolarization in motor neurons during pilocarpine evoked CPG activity relates to the tonic depolarization observed in motor neurons during treadmill stepping [40, 74]. Under this assumption, we visualized in our experiments an important determinant of a motor neuron’s depolarization during walking, that is [Ca^2+^]_i_.

By which mechanism Ca^2+^ affects the membrane potential is yet unclear. Ca^2+^ may act directly as a charge carrier as it has been shown for ligand-gated ACh currents in the motor neurons [58]. Ca^2+^ as a charge carrier entering motor neurons through voltage-gated channels has been shown in different vertebrates [75–77] and a motor neuron in cockroaches [66]. In all these studies, however, Ca^2+^ has been shown to support plateau potentials, which have so far not been observed in stick insects [26, 45]. Interestingly, in *Drosophila* motor neurons L-type Ca^2+^ currents do not support a persistent inward current but they enhance motor neuron excitability through interaction with other membrane currents [78]. Ca^2+^ currents may play a similar role in stick insect motor neurons.

### Ca^2+^ oscillations in DUM neurons

In insects, efferent thoracic DUM neurons are local octopaminergic neurons that primarily innervate skeletal muscles [34–37] and modulate muscle contractions [38, 39]. In the stick insect the lateral nerve nl5 contains the axons of 5–6 dorsal unpaired median (DUM) neurons [22]. In all DUM neurons recorded here, oscillations in [Ca^2+^]_i_ were observed in the presence of pilocarpine. Oscillation periods were 40 to 68% longer than oscillation periods in RetCx MNs and there was no indication of coupling between DUM neurons and RetCx MNs. Both observations indicate that the driving sources of both neuron types are independent.

However, we cannot exclude that DUM neurons are coupled to the activity in motor neurons controlling movements of other leg joints which oscillate independently from RetCx MNs [41]. Coupling of DUM neuron activity to pilocarpine evoked as levator MN activity has been observed in *Manduca sexta* [79] and sometimes in the locust for the metathoracic DUM (3,4,5), indicating a common drive of these motor and DUM neurons [80].

Using reset experiments, Baudoux et al. [80] did not find indications that DUM neurons are parts of the rhythm generating networks. Also, DUM neuron cell bodies from locusts do not appear to be endogenous bursters when treated with pilocarpine [81]. Therefore, we assume that also in stick insects an external source provides rhythmic drive to DUM neurons. Pilocarpine also evokes rhythmic activity in thoracic DUM neurons in the isolated nervous system of larvae of *Manduca* [79]. These DUM neurons are synchronously depolarized, i.e. in phase, partially due to input from the subesophageal ganglion. In contrast, in the presence of pilocarpine, rhythmic activity of different stick insect DUM neurons is not necessarily phase coupled, indicating independent central rhythmic drive.

In summary, our data indicate that pilocarpine activates different CPGs that cause rhythmic activity in mesothoracic RetCx MNs and mesothoracic efferent DUM neurons independently of each other. In addition, the activities in at least three of the DUM neurons appear to be also structured by inputs from CPGs that oscillate independently of each other.

In contrast to pilocarpine-evoked activity, during single middle leg stepping, mesothoracic DUM neurons are synchronously activated in constant phase with motor neuron activity that is in the late stance phase of a step [62]. Possibly, during leg stepping, sense organs cause synchronization of the activity in the leg’s DUM neurons and the coupling to motor neuron activity. This conclusion derives from accumulating evidence that in a walking stick insect, the coordination of rhythmic activity patterns in different motor neurons [40, 82] results from the interaction of central pattern generating networks and leg sense organs [24, 40].

### Concluding remarks

The preparation introduced in this study is well suited for using changes in [Ca^2+^]_i_ as a proxy for efferent neuron activity in the stick insect. The Ca^2+^ oscillations in motor neurons evoked by pharmacological activation of CPGs appear to mimic changes in [Ca^2+^]_i_ in these neurons during walking. For gaining further insights into the specific role of Ca^2+^ in efferent neurons during walking, a modified version of the set-up should allow [Ca^2+^]_i_ measurements in single efferent neurons during leg stepping.

## Supporting information

S1 Data Calcium integral over burst integral. Raw Data for Fig 2C. Normalized data in the green field were used to calculate regressions in Matlab. See also Material and method section.
(XLSX)

S2 Data Data from intracellular recordings of RetCx MNs with QX 314 electrodes. (XLSX)

S3 Data Oscillation periods. Raw Data for Fig 6. Experiment 09.10.12 T2 pilo3x10-3_1 nl2 nl5.
(XLSX)

S4 Data DUM and RetCx MN oscillation periods. (XLSX)

S1 Fig Intracellular recording of a retractor motor neuron‘s membrane potential. Even after 14 minutes of recording time, action potentials could be evoked by depolarizing current injection into the motor neuron (2nd trace). Each action potential measured by the intracellular electrode (1st trace) was picked up by the extracellular electrode at nerve nl5. Nerve nl5 contains the axons of RetCx MNs.
(TIF)
