# Supplementary figures and images for: Calcium imaging of CPG-evoked activity in efferent neurons of the stick insect
Source: PLoS One. 2018 Aug 24;13(8):e0202822. doi: 10.1371/journal.pone.0202822 (PMC6108493; doi:10.1371/journal.pone.0202822)

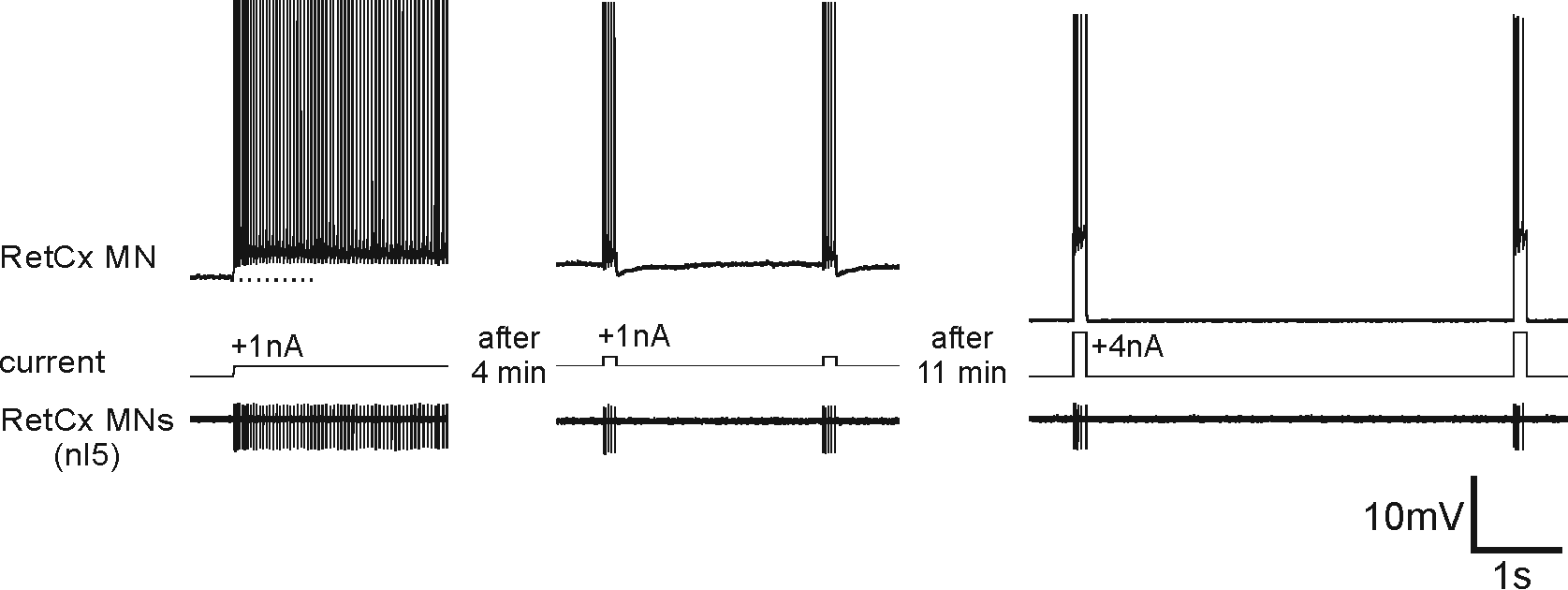

Supplement: S1 Fig — Even after 14 minutes of recording time, action potentials could be evoked by depolarizing current injection into the motor neuron (2nd trace). Each action potential measured by the intracellular electrode (1st trace) was picked up by the extracellular electrode at nerve nl5. Nerve nl5 contains the axons of RetCx MNs. (TIF) [file pone.0202822.s005.tif]
